# HuoXue QianYang QuTan recipe attenuates myocardial hypertrophy in obese hypertensive rats by regulating MPC1/MCT4 mediated pyruvate-lactate metabolic axis

**DOI:** 10.1186/s13020-025-01240-9

**Published:** 2025-10-23

**Authors:** Jing Wang, Meng Wang, Jianhua Li, Da Li, Deyu Fu

**Affiliations:** 1 Department of Traditional Chinese Medicine, Huadong Hospital, Fudan University, 221 Yan’an West Road, Jing’an District, Shanghai, 200040 People’s Republic of China; 2 Department of Nephrology, Shuguang Hospital Affiliated to Shanghai University of Traditional Chinese Medicine, Shanghai, 201203 China; 3 Key Laboratory of Liver and Kidney Diseases, Ministry of Education, Shanghai University of Traditional Chinese Medicine, Shanghai, 201203 China; 4 TCM Institute of Kidney Disease, Shanghai University of Traditional Chinese Medicine, Shanghai, 201203 China; 5 Shanghai Key Laboratory of Traditional Chinese Clinical Medicine, Shanghai University of Traditional Chinese Medicine, Shanghai, 201203 China; 6 Department of Cardiology, Yueyang Hospital of Integrated Traditional Chinese and Western Medicine, Shanghai University of Traditional Chinese Medicine, 110 Ganhe Road, Hongkou District, Shanghai, 200136 People’s Republic of China

**Keywords:** Glycolysis, Pyruvate-lactate metabolism, Myocardial hypertrophy, Obesity-related hypertension, Traditional Chinese medicine

## Abstract

**Background:**

HuoXue QianYang QuTan Recipe (HQQR) is an effective prescription for the clinical management of myocardial hypertrophy in patients with obesity-related hypertension (OBH). In previous studies, HQQR showed the pharmacological properties of mitochondrial protection, anti-inflammatory, and anti-oxidant in OBH rats, which were closely related to its cardioprotective effects. This study is designed to further explore the molecular mechanisms by which HQQR attenuates myocardial hypertrophy in OBH rats.

**Methods:**

High-fat diet-fed SHR were gavaged with HQQR (5 ml/kg or 10 ml/kg, 3.87 g/ml of original drug) or Valsartan (10 ml/kg, 3 mg/ml of original drug) for 10 weeks. In vitro, the H9C2 cells were stimulated with angiotensin-II (Ang-II) and (or) free fatty acid (FFA) in the absence or presence of HQQR. The effects of HQQR on lipid metabolism, mitochondrial function, glycolysis, pyruvate-lactate axis, and myocardial hypertrophy were examined by immunoblotting, pathological analysis, WGA, oil red O and colorimetric methods. Next, to further explore how HQQR attenuates myocardial hypertrophy, we transfected MPC1 siRNA or MCT4 siRNA into H9C2 cells. Finally, the lactate-stimulated H9C2 cells were treated with HQQR and (or) VB124 (a lactate transporter inhibitor), and the degree of myocardial hypertrophy was evaluated by immunoblotting.

**Results:**

Compared to SHR rats, OBH rats exhibited more pronounced blood pressure, lipid metabolism disorder and myocardial pathology injury, which was attenuated by HQQR treatment. Furthermore, HQQR significantly improved the mitochondrial function, glycolysis, and pyruvate-lactate metabolic axis in OBH rats and Ang-II + FFA-treated H9C2 cells, thereby attenuating myocardial hypertrophy. However, these protective effects of HQQR were attenuated in H9C2 cells transfected with MPC1 siRNA and enhanced in cells transfected with MCT4 siRNA. Next, we found that HQQR dose-dependently inhibited the expression β-MHC and ANP proteins in lactate-stimulated H9C2 cells, and that this effect was further enhanced by combination with VB124.

**Conclusion:**

HQQR inhibits glycolysis and restores pyruvate-lactate metabolism in OBH rats by regulating MPC1/MCT4, thereby attenuating myocardial hypertrophy.

**Supplementary Information:**

The online version contains supplementary material available at 10.1186/s13020-025-01240-9.

## Introduction

The number of adults with hypertension is increasing globally, and overweight and obesity have become one of the main causes of the rising incidence of hypertension [1, 2]. Myocardial hypertrophy, one of the characteristic manifestations of myocardial remodelling, is a well-recognised marker of hypertensive target organ damage, while obesity and its associated metabolic dysfunction diminish the protective effect of antihypertensive drugs [3, 4]. The study found that obesity hypertension (OBH) patients have a higher risk of developing myocardial hypertrophy and a more aggressive condition, which is an urgent therapeutic challenge to be solved [5].

The heart is a high-energy-demanding organ, with approximately 90% of its energy supply dependent on mitochondrial oxidative phosphorylation [6]. Pathological myocardial hypertrophy is often accompanied by changes in energy metabolism, as evidenced by increased glucose metabolism and decreased fatty acid metabolism [7]. Although this metabolic shift is a protective compensatory response, glycolysis and pyruvate oxidation become decoupled under pathological conditions, manifested by a reduced rate of pyruvate entry into the mitochondria alongside an increased rate of its conversion to lactate, ultimately leading to enhanced lactate efflux [8]. Sansbury and colleagues have found that in myocardial hypertrophy, the lactate/pyruvate ratio of the damaged myocardium reaches 2.5 times that of normal myocardium despite an elevated rate of glucose metabolism, suggesting an increase in anaerobic metabolism [9]. Ni et al. reported that ginsenoside Rg3 attenuated myocardial hypertrophy by increasing myocardial pyruvate oxidation [10]. Thus, pyruvate-lactate metabolic imbalance is a pathological hallmark of myocardial hypertrophy and a key therapeutic target. Mitochondrial pyruvate carrier 1 (MPC1) and monocarboxylate transporter 4 (MCT4) are key molecular switches that regulate pyruvate-lactate axis homeostasis in cardiomyocytes, where MPC1 mediates the transfer of pyruvate to mitochondria for oxidation and MCT4 mediates cellular lactate shuttling [11–13]. Growing evidence suggests that restoration of the pyruvate-lactate metabolism through MPC1 up-regulation and MCT4 inhibition is key to attenuating myocardial hypertrophy [14, 15].

Numerous clinical and experimental studies have also confirmed the efficacy of traditional Chinese herbal formulas in treating myocardial hypertrophy [16, 17]. HuoXue QianYang QuTan Recipe (HQQR) is an effective prescription for the prevention and treatment of myocardial hypertrophy in OBH based on the theory of “blood stasis, hyperactivity of yang, and phlegm turbidity” from the Yellow Emperor’s Inner Canon (Huang Di Nei Jing). As the main drug in HQQR, *Salvia miltiorrhiza* Bge. and its active ingredients, such as salvianolic acids, cryptotanshinone, rosmarinic acid, have been found to significantly reduce myocardial injury through anti-inflammation, anti-oxidation, and restoration of mitochondrial energy metabolism [18–21]. Li et al. reported ferulic acid attenuated stress-induced myocardial injury by activating the PPARα/PGC-1α and Nrf2 metabolic pathways [22]. Moreover, previous studies have demonstrated that ligustilide, betaine, luteolin and chlorogenic acid exhibit cardioprotective effects by reducing myocardial hypertrophy across multiple disease models [23–26]. Clinically, HQQR can reverse myocardial hypertrophy in OBH patients by improving glycolipid metabolism and lowering blood pressure [27]. Furthermore, animal studies have demonstrated that HQQR significantly increases mitochondrial copy number, ATP content, and respiratory chain activity and improves mitochondrial morphology in cardiomyocytes by SIRT1/PGC-1α deacetylation pathway, thereby reducing myocardial hypertrophy in OBH rats [28]. MPC1/MCT4 have been recognised as molecules closely associated with mitochondrial morphology and function, thereby influencing the progression of myocardial hypertrophy [6, 29]. Li et al. reported that PGC-1α enhanced MPC1 expression to promote pyruvate oxidation and improve mitochondrial function [30]. Meanwhile, Ma et al. discovered that overexpression of MCT4 mediated fatty acid-induced cardiomyocyte injury by disrupting mitochondrial metabolism [31]. Building upon our previous findings, this investigation aimed to further elucidate the mechanisms of HQQR against myocardial hypertrophy in OBH rats.

## Materials and methods

### Animals and medicines

The experimental protocol involving animals received full ethical approval from the Institutional Animal Care and Use Committee of Shanghai University of TCM (Approval No. PZSHUTCM2404280007). Male spontaneously hypertensive rats (SHR, 5 week-old) and age/sex-matched WKY rats were purchased from the Vital River Co., Ltd (Beijing, China). The rats were acclimatized in climate-controlled isolator cages and all procedures complied with humane animal testing guidelines. HQQR contains *Salvia miltiorrhiza* Bge., *Ligusticum chuanxiong* Hort., *Uncaria rhynchophylla* (Miq.) Miq. ex Havil., *Haliotis diversicolor* Reeve, *Taxillus chinensis* (DC.) Danser, *Crataegus pinnatifida* Bge. and *Zea mays* L. The detailed information on HQQR can be viewed in Supplementary Table 1 and the original drugs were purchased from Shanghai WanShiCheng Pharmaceutical Co. Ltd. Valsartan capsules were obtained from Novartis Pharmaceutical Co., Ltd and used as a positive control.

### Reagents and antibodies

The primary antibodies included anti-β-myosin heavy chain (β-MHC, A22140, Abclonal, China), anti-atrial natriuretic peptide (ANP, A14755, Abclonal, China), anti-lactate dehydrogenase (LDHA, ab101562, Abcam, UK), anti-MPC1 (PS13040S, Abmart, China), anti-MCT4 (TD4182S, Abmart, China), anti-Mn-SOD (AB68155, Abcam, UK), anti-ATP6 (A8193, Abclonal, China), anti-p-PKM (#3827S, CST, USA), anti-glucose transporter-1 (GLUT1, A11727, Abclonal, China), anti-GLUT4 (PA3318S, Abmart, China), anti-hexokinase 2 (HK2, ab209847, abcam, UK), anti-GAPDH (60004-1-Ig, Proteintech, China) and anti-α-tubulin (66031-1-Ig, Proteintech, China). The fluorescent probe for rhodamine-labelled phalloidin (C2207S) was purchased from Beyotime (Shanghai, China). The angiotensin II (Ang-II, HY-13948) and VB124 (HY-139665) were purchased from MCE (USA). The pyruvate (A081-1-1) and lactate (A019-2-1) kit were from Jiancheng Institute (Nanjing, China). The lactate (L1750) was provided by Sigma (Saint Louis, MO). The rapid blocking buffer (PS108P) and ECL kit (SQ201) were from Yamei Biotechnology Co., Ltd (Shanghai, China).

### Preparation of HQQR and identification of the main chemical components

The aqueous extract of HQQR was prepared following our laboratory's optimized decoction protocol. The herbs contained in HQQR were mixed in proportion, crushed into small particles, and then boiled twice under continuous stirring. The aqueous extract was distilled and concentrated to approximately 3.87g (raw herbal product)/mL, and finally filtered. The chemical compositions of the aqueous extract were identified by Luming Biotech CO., Ltd. (Shanghai, China) using the ultra-high performance liquid chromatography-mass spectrometry (UPLC-MS) analysis. The column was an ACQUITY UPLC HSS T3 (100 mm × 2.1 mm, 1.8 μm) with a column temperature of 45 ℃. Mobile phase A was 0.1% formic acid–water, mobile phase B was acetonitrile, the flow rate was 0.35 mL/min, and the PDA scan range was 210–400 nm. The analysis was performed strictly according to the set elution gradient. According to the set mass spectrometry parameters, the mass spectrometry signal of the sample was collected using both positive ion and negative ion scanning modes.

### HQQR-medicated serum preparation

Twenty male SD rats (220 ± 20 g) were selected for this study. These rats were then divided into two groups: an HQQR group and a control group, 10 rats in each group. Each rat was gavaged with 0.9% normal saline or HQQR solution (3.87 g/mL of drug) at a dose of 10 mL/kg twice a day for 3 consecutive days, respectively. Two hours after the final administration, rats were anaesthetised with sodium pentobarbital (40 mg/kg, i.p.). Blood was subsequently collected from the abdominal aorta and centrifuged to isolate serum. The serum was heat-inactivated in a 56 ℃ water bath for 30 min and sterilized by filtration through a 0.22 μm needle filter.

### Animal model

The OBH rat model was prepared by feeding SHR rats with high-fat diet (HFD), and age/sex-matched WKY rats were used as controls. The high-fat feeds were obtained from Fanbo Co., Ltd. (Shanghai, China) and consisted of 60% normal feed, 4.7% raw peanut, 10% yolk powder, 1% sesame oil, 12% lard, 2% salt, 5% sucrose, 2% cholesterol, 3% milk powder, and 0.3% bile salt. After 10 weeks, rats in the top 1/3 of body weight were identified as OBH rats based on previous reports [32]. Next, the OBH rats were randomly divided into 4 groups (*n* = 8) and continued to be fed with HFD: (1) OBH-HF group, (2) OBH-HF/L group (5 ml/kg of HQQR, containing 3.87 g/ml of original drug), (3) OBH-HF/H group (10 ml/kg of HQQR), (4) OBH-HF/V group (10 ml/kg of Valsartan, containing 3 mg/ml of original drug). The other 8 SHR and WKY rats were divided into SHR-ND and WKY-ND groups, respectively, and fed normal diet. Each rat was gavaged once daily for 10 weeks.

### Cell culture

The H9C2 cells, a rat cardiomyocyte, were purchased from the Pricella Co., Ltd (Wuhan, China) and cultured in DMEM supplemented with 10% FBS. To model myocardial hypertrophy, 85% confluence of H9C2 cells were treated with 1 μM of Ang-II and (or) free fatty acid (FFA) at 37 ℃ with 5% CO_2_ for 24 h. In this case, the cells were treated with or without HQQR-medicated serum. To observe the effect of lactate on cardiomyocytes, the H9C2 cells were treated with different concentrations of lactate (0.5 μM, 2 μM, 8 μM) with or without HQQR-medicated serum.

### Western blot assay

Both myocardial tissues and H9C2 cells were lysed using RIPA buffer, and protein concentrations were quantified via the BCA assay. Proteins were separated electrophoretically on 8% SDS-PAGE gels and transferred to PVDF membranes. After sealing with a quick sealer, the PVDF membranes were incubated with primary antibodies according to the instructions. After incubation with secondary antibody to amplify the signal, the signal was visualised with ECL solution.

### Pathology and oil red O staining

The 4 μm sections from the paraffin-embedded heart tissue were performed hematoxylin & eosin (HE) and Masson staining according to the standard protocol. For Oil Red O staining, OCT-embedded frozen sections were immersed in 60% isopropanol for 30 s and then incubated with Oil Red O staining solution for 15 min. Next, sections were placed in 60% isopropanol for colour separation, followed by a drop of Mayer's Hematoxylin staining solution for 2 min to stain the nuclei. Cardiac lipid droplets were observed under a light microscope at 200 × magnification and subjected to quantitative analysis using Image J software.

### Wheat Germ Agglutinin (WGA) and phalloidin staining

Paraffin-embedded sections were sequentially deparaffinized in xylene and absolute ethanol, followed by antigen retrieval in EDTA buffer. Sections were incubated with WGA staining solution for 1 h at 37 ℃ protected from light, then counterstained with DAPI for nuclei visualization. For phalloidin staining, H9C2 cells were seeded evenly in six-well plates. After treatment, the medium was removed and cells were fixed with 4% paraformaldehyde. Following washes, cells were incubated with 1% phalloidin working solution for 30 min in the dark. Cardiomyocyte morphology was visualized using fluorescence microscopy at 200 × or 400 × magnification.

### Determination of lactate/pyruvate ratio

The ratio of lactate to pyruvate was determined by the colourimetric method according to the instructions. Briefly, the serum was obtained by centrifuging the blood at 4 ℃ for 15 min. The serum was diluted in specific proportions and mixed with the test solution, then the mixture was reacted for 10 min at 37 ℃. Next, the mixture was incubated with the colour development solution for 10 min at room temperature. The absorbance values of lactate and pyruvate were measured at 530 nm and 505 nm wavelengths, respectively. The concentrations of lactate and pyruvate were calculated from the standard curves.

### FFA determination

At the end of the intervention, blood was collected from the abdominal aorta of the rats and centrifuged to obtain serum. Serum FFA levels were tested by an automated biochemical analyser.

### siRNA transfection

Rat MPC1 siRNA (5′-GCAAAGCAGCGGACUAUGUTT-3′) and MCT4 siRNA (5′-CUCAAUCGAUACUUCAACATT-3′) were synthesised by GenePharma (Shanghai, China). Negative control or target siRNA was transfected into H9C2 cells using lipofectamine 3000 reagent according to the manufacturer’s protocol. Briefly, cells were seeded in six-well plates to achieve 60–70% confluence. Lipid-siRNA complexes were prepared and added to cells for 12 h, after which the medium was replaced with fresh medium for subsequent treatments.

### Statistical analysis

All experimental data were expressed as mean ± SD. Statistical significance was determined using ANOVA followed by LSD post hoc test for multiple comparisons, implemented in IBM SPSS Statistics 25.0 (IBM Corp., Armonk, NY, USA). A probability threshold of *p* ≤ 0.05 was applied to define statistical significance.

## Results

### Component analysis of HQQR

**Fig. 1:**
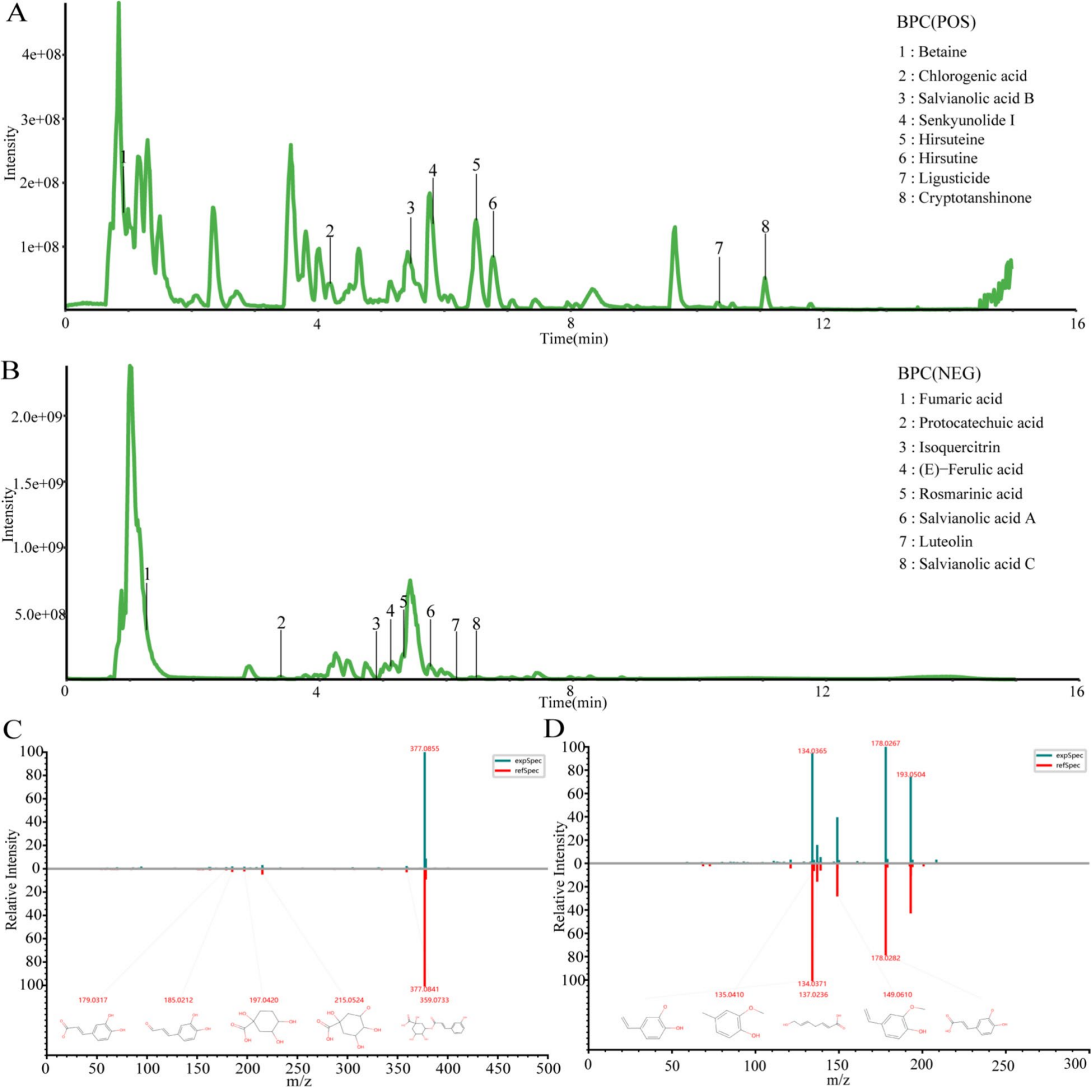
Component analysis of HQQR. UPLC-MS was used to analyse the chemical composition of HQQR and obtain the Base-peak chromatogram (BPC) of HQQR. **A** Scan with positive ions (HQQR sample). **B** Scan with negative ions (HQQR sample). **C** Ion chromatogram of chlorogenic acid. **D** Ion chromatogram of ferulic acid

In the current study, we first used UPLC-MS to analyse the chemical composition of HQQR. A total of 912 chemical components were identified (Supplementary Table 2), among which 16 representative natural products were displayed in the total ion chromatogram in either positive BPC mode (Fig. 1A) or negative BPC mode (Fig. 1B), including Betaine, Chlorogenic acid, Salvianolic acid B, Senkyunolide I, Hirsuteine, Hirsutine Ligusticide, Cryptotanshinone, Fumaric acid, Protocatechuic acid, Isoquercitrin, (E)-Ferulic acid, Rosmarinic acid, Salvianolic acid A, Luteolin, Salvianolic acid C. Meanwhile, the ion chromatograms of chlorogenic acid and ferulic acid were shown in Fig. 1C and Fig. 1D, respectively, and the ion chromatograms of the other 14 representative components were shown in Supplementary Fig. 1A–N. In addition, we classified the identified compounds and found that phenylpropanoids, carbohydrates and glycosides, flavonoids and terpenes were more abundant in HQQR (Supplementary Fig. 2).

### HQQR improves lipid metabolism and cardiomyocyte injury in OBH rats

**Fig. 2:**
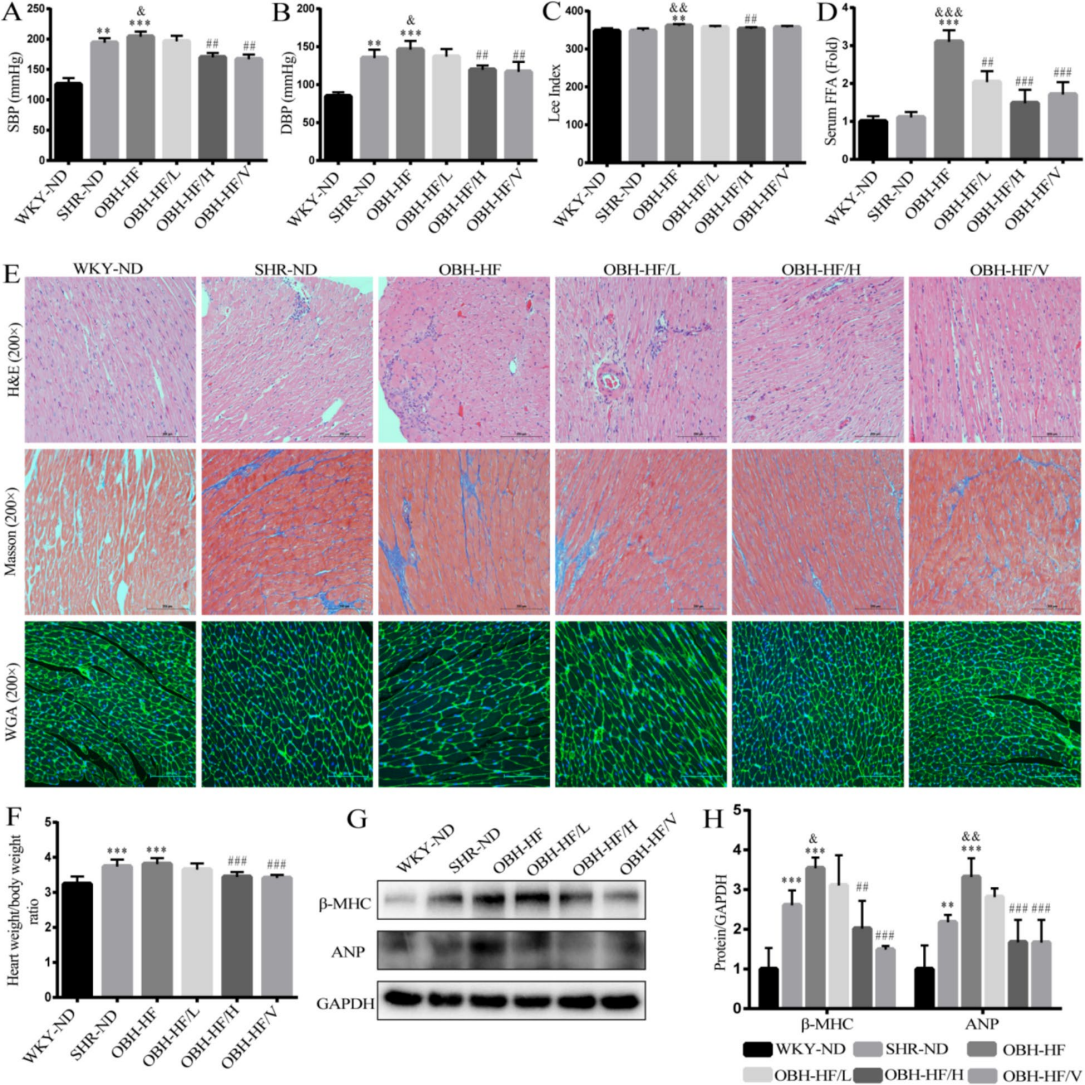
HQQR attenuates lipid metabolism and cardiomyocyte injury in OBH rats. The SHR were fed on HFD for 10 weeks to establish the OBH model. The OBH rats then were gavaged with HQQR (OBH-HF/L or OBH-HF/H) or Valsartan (OBH-HF/V) for 10 weeks. The systolic blood pressure (**A**), diastolic blood pressure (**B**), Lee index (**C**), and serum FFA (**D**) were measured in rats (*n* = 8). **E** Myocardial histopathology and degree of hypertrophy were assessed by H&E, Masson, and WGA. **F** Measurement of heart weight/body weight ratio (*n* = 8). **G** The β-MHC and ANP protein expression in myocardial tissue from each group of rats was tested by immunoblotting. **H** Quantitative analysis of β-MHC and ANP protein levels in myocardial tissue of rats (*n* = 4). ^**^p < 0.01, ^***^p < 0.001 *vs.* WKY-ND group, ^&^*p* < 0.05, ^&&^*p* < 0.01 *vs.* SHR-ND group, ^##^*p* < 0.01, ^###^*p* < 0.001 *vs.* OBH-HF group

Firstly, in the present study, we evaluated the effects of HQQR on blood pressure and serum biochemistry in OBH rats. Our results demonstrated that HFD administration markedly elevated the blood pressure, Lee index and FFA levels compared to SHR rats, which was attenuated by HQQR (Fig. 2A–D). Next, we performed the HE and Masson staining to evaluate cardiomyocyte injury and fibrosis. We found that myocardial tissue from SHR rats exhibited significant injury, inflammatory cell infiltration and fibrosis, which was further enhanced by combination with HFD (Fig. 2E). In addition, WGA staining, a fluorescent dye that specifically binds to glycoproteins on cardiomyocyte membranes, showed more pronounced myocardial hypertrophy in the OBH-HF group compared to the SHR-ND group (Fig. 2E). Of note, treatment with HQQR for 10 weeks ameliorated the myocardial hypertrophy and pathological injury in OBH rats (Fig. 2E). Moreover, the heart weight/body weight ratio increased in both the OBH-HF and SHR-ND groups, whereas HQQR treatment reduced this parameter (Fig. 2F). Next, we determined the expression of markers associated with myocardial hypertrophy. Immunoblotting revealed increased expression of β-MHC and ANP proteins in the SHR-ND group compared to the WKY-ND group (Fig. 2G and H). More importantly, HFD treatment further elevated the levels of β-MHC and ANP proteins compared to SHR-ND group, which was reversed by HQQR administration (Fig. 2G and H). These results identify that HQQR achieves the cardioprotective effects in OBH rats.

### HQQR improves Ang-II combined with FFA induced hypertrophy in H9C2 cells

**Fig. 3:**
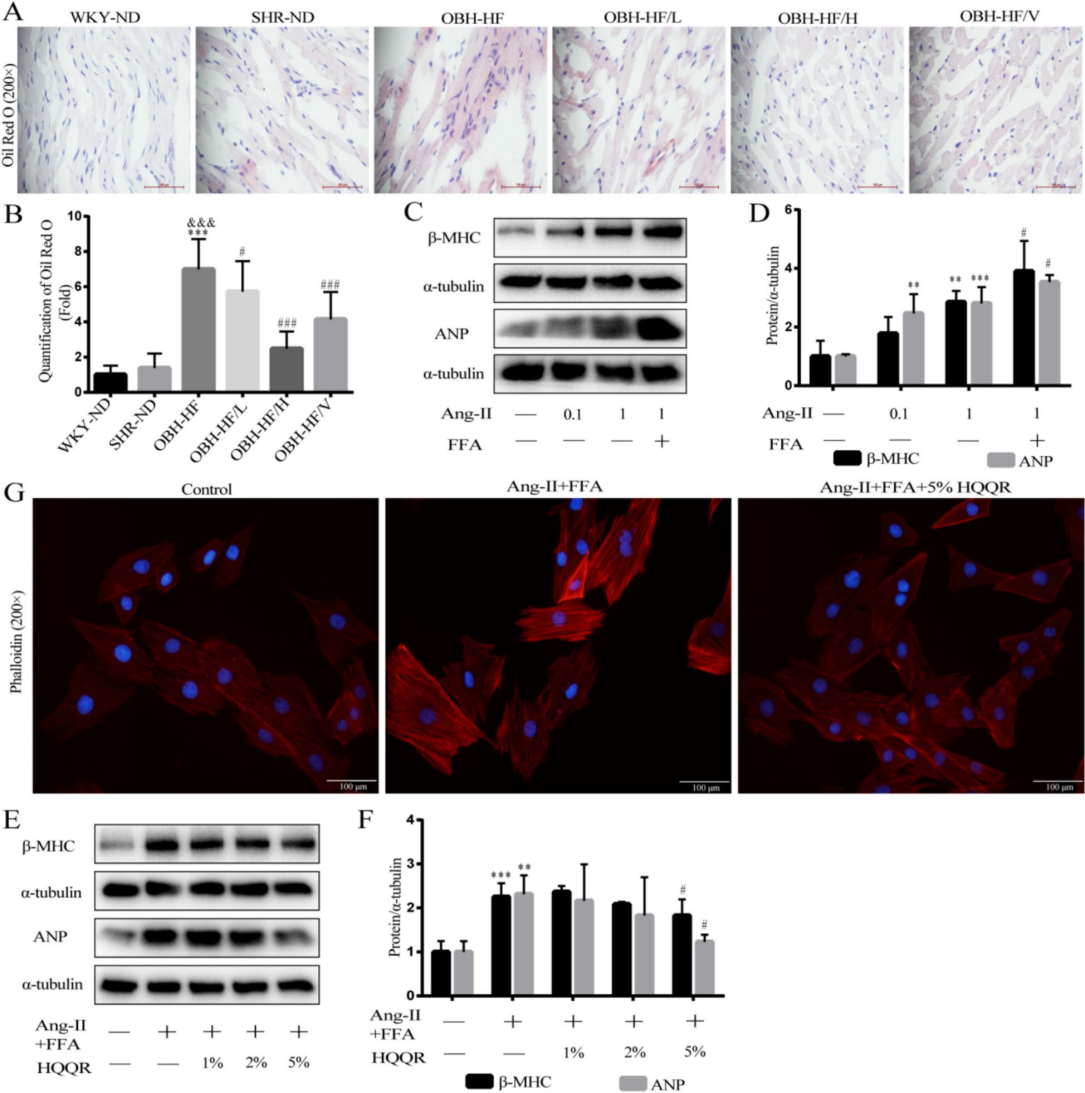
HQQR improves Ang-II combined with FFA induced hypertrophy in H9C2 cells. **A** Lipid droplet deposition in cardiomyocytes of rats was observed by oil red O staining. **B** Quantitative analysis of Oil Red O staining (*n* = 3). **C** Representative immunoblot demonstrating β-MHC and ANP protein expression in H9C2 cells under different treatments. **D** Quantitative analysis of β-MHC and ANP proteins in H9C2 cells (*n* = 3). **E** Representative immunoblot demonstrating β-MHC and ANP protein expression in H9C2 cells under different treatments. **F** Quantitative analysis of β-MHC and ANP proteins in H9C2 cells (*n* = 4). **G** The morphology of H9C2 cells was observed by phalloidin staining using fluorescence microscopy at 200 × magnification. ^**^p < 0.01, ^***^p < 0.001 *vs.* control group, ^#^*p* < 0.05 *vs.* Ang-II (1μM) or Ang-Ⅱ + FFA group

Oil red O results revealed the increased lipid accumulation in the cardiomyocytes of the OBH rats compared with the SHR rats, which was attenuated after HQQR treatment (Fig. 3A and B). To further assess the effects of lipid accumulation on myocardial hypertrophy, H9C2 cells were treated with Ang-II and (or) FFA. Immunoblotting showed that Ang-II combined with FFA markedly increased the levels of ANP and β-MHC proteins compared with Ang-II stimulation alone (Fig. 3C and D). Furthermore, immunoblotting showed that HQQR-medicated serum concentration-dependently decreased the levels of β-MHC and ANP proteins in Ang-II + FFA-treated H9C2 cells (Fig. 3E and F). Next, we used the phalloidin fluorescent dye to specifically label the F-actin of cardiomyocytes. Our results demonstrated that stimulation with Ang-II + FFA significantly induced the cardiomyocyte hypertrophy, which was inhibited by HQQR treatment (Fig. 3G).

### HQQR attenuates mitochondrial dysfunction, glycolysis and lactate/pyruvate ratio in both OBH rats and H9C2 cells

**Fig. 4:**
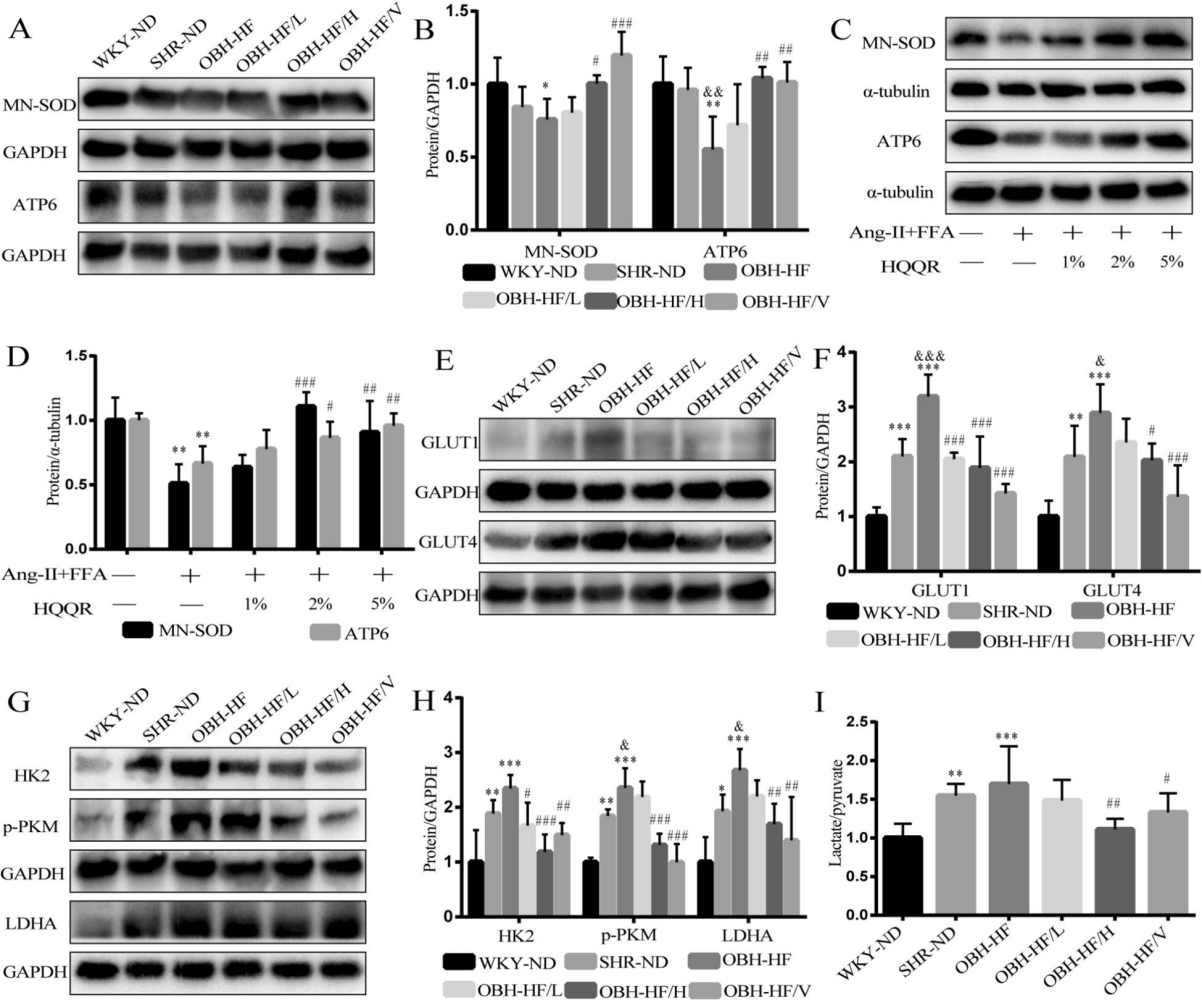
HQQR attenuates mitochondrial dysfunction, glycolysis and lactate/pyruvate ratio in both OBH rats and H9C2 cells. **A** The Mn-SOD and ATP6 protein expression in myocardial tissue from each group of rats was tested by immunoblotting. **B** Quantitative analysis of Mn-SOD and ATP6 protein levels in myocardial tissue of rats (*n* = 4). **C** Representative immunoblot demonstrating Mn-SOD and ATP6 protein expression in H9C2 cells under different treatments. **D** Quantitative analysis of Mn-SOD and ATP6 proteins in H9C2 cells (*n* = 4). **E** The GLUT1 and GLUT4 protein expression in myocardial tissue from each group of rats was tested by immunoblotting. **F** Quantitative analysis of GLUT1 and GLUT4 protein levels in myocardial tissue of rats (*n* = 4). **G** The HK2, p-PKM and LDHA protein expression in myocardial tissue from each group of rats was tested by immunoblotting. **H** Quantitative analysis of HK2, p-PKM and LDHA protein levels in myocardial tissue of rats (*n* = 4). **I** Serum lactate and pyruvate levels were measured in rats and then the lactate/pyruvate ratio was calculated. ^*^p < 0.05, ^**^p < 0.01, ^***^p < 0.001 *vs.* WKY-ND or control group, ^&^*p* < 0.05, ^&&^*p* < 0.01, ^&&&^*p* < 0.001 *vs.* SHR-ND group, ^#^*p* < 0.05, ^##^*p* < 0.01, ^###^*p* < 0.001 *vs.* OBH-HF or Ang-II + FFA group

We have found that HQQR increases mitochondrial biosynthesis and ATP production in OBH rats [28]. This study further demonstrated that HQQR treatment increased Mn-SOD and ATP6 protein levels in OBH rats and Ang-II + FFA-treated H9C2 cells, indicating the mitochondrial protective effect (Fig. 4A–D). Mitochondrial dysfunction positively correlates with activation of glycolysis. To further assess the effect of HQQR on glucose metabolism, we examined the expression of glycolysis-related proteins. As shown in Fig. 4E and F, the expression of GLUT1 and GLUT4 proteins was significantly increased in SHR-ND group compared to WKY-ND group, which was further enhanced in the OBH-HF group. In addition, HK2, p-PKM and LDHA proteins, the key proteins in glycolysis, were increased in SHR hearts compared to the WKY-ND group, and HFD administration further accelerated the rate of glycolysis (Fig. 4G and H). Conversely, treatment with HQQR for 10 weeks decreased the content of glucose transport and glycolysis proteins in OBH rats (Fig. 4E–H). To further determine whether mitochondrial injury and enhanced glycolysis disrupt the pyruvate/lactate metabolic axis, we examined the lactate/pyruvate ratio in serum. The lactate/pyruvate ratio was elevated in SHR rats compared to WKY-ND group, and HFD made this ratio higher, suggesting that pyruvate is converted more to lactate than to mitochondrial oxidation (Fig. 4I). Of note, HQQR improved the lactate/pyruvate metabolic axis in OBH rats (Fig. 4I).

**Fig. 5:**
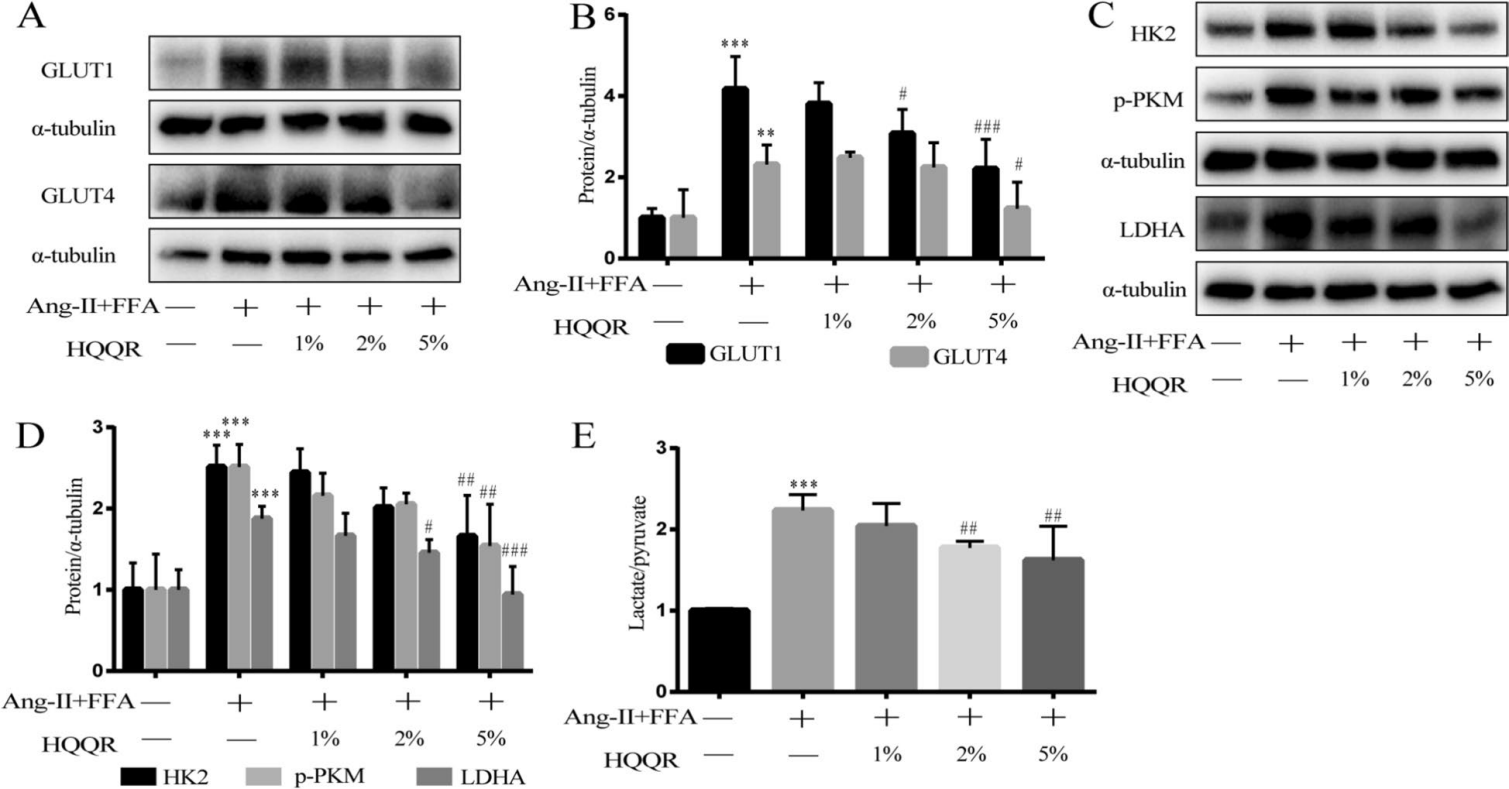
HQQR attenuates mitochondrial dysfunction, glycolysis and lactate/pyruvate ratio in both OBH rats and H9C2 cells. **A** Representative immunoblot demonstrating GLUT1 and GLUT4 protein expression in H9C2 cells under different treatments. **B** Quantitative analysis of GLUT1 and GLUT4 proteins in H9C2 cells (*n* = 3). **C** Representative immunoblot demonstrating HK2, p-PKM and LDHA protein expression in H9C2 cells under different treatments. **D** Quantitative analysis of HK2, p-PKM and LDHA proteins in H9C2 cells (*n* = 4). **E** Lactate and pyruvate levels in H9C2 medium were measured after the treatments in each group and the lactate/pyruvate ratio was calculated. ^**^p < 0.01, ^***^p < 0.001 *vs.* control group, ^#^*p* < 0.05, ^##^*p* < 0.01, ^###^*p* < 0.001 *vs.* Ang-II + FFA group

Furthermore, immunoblotting demonstrated that FFA combined with Ang-II increased the levels of HK2, p-PKM and LDHA proteins compared to Ang-II treatment alone (Supplementary Fig. 3). More importantly, treatment with HQQR inhibited the expression of glucose transport proteins and glycolysis-related proteins and decreased the ration of lactate/pyruvate in a concentration-dependent manner (Fig. 5A–E).

### HQQR improves pyruvate-lactate metabolism and myocardial hypertrophy in vivo and in vitro by targeting MPC1/MCT4

**Fig. 6:**
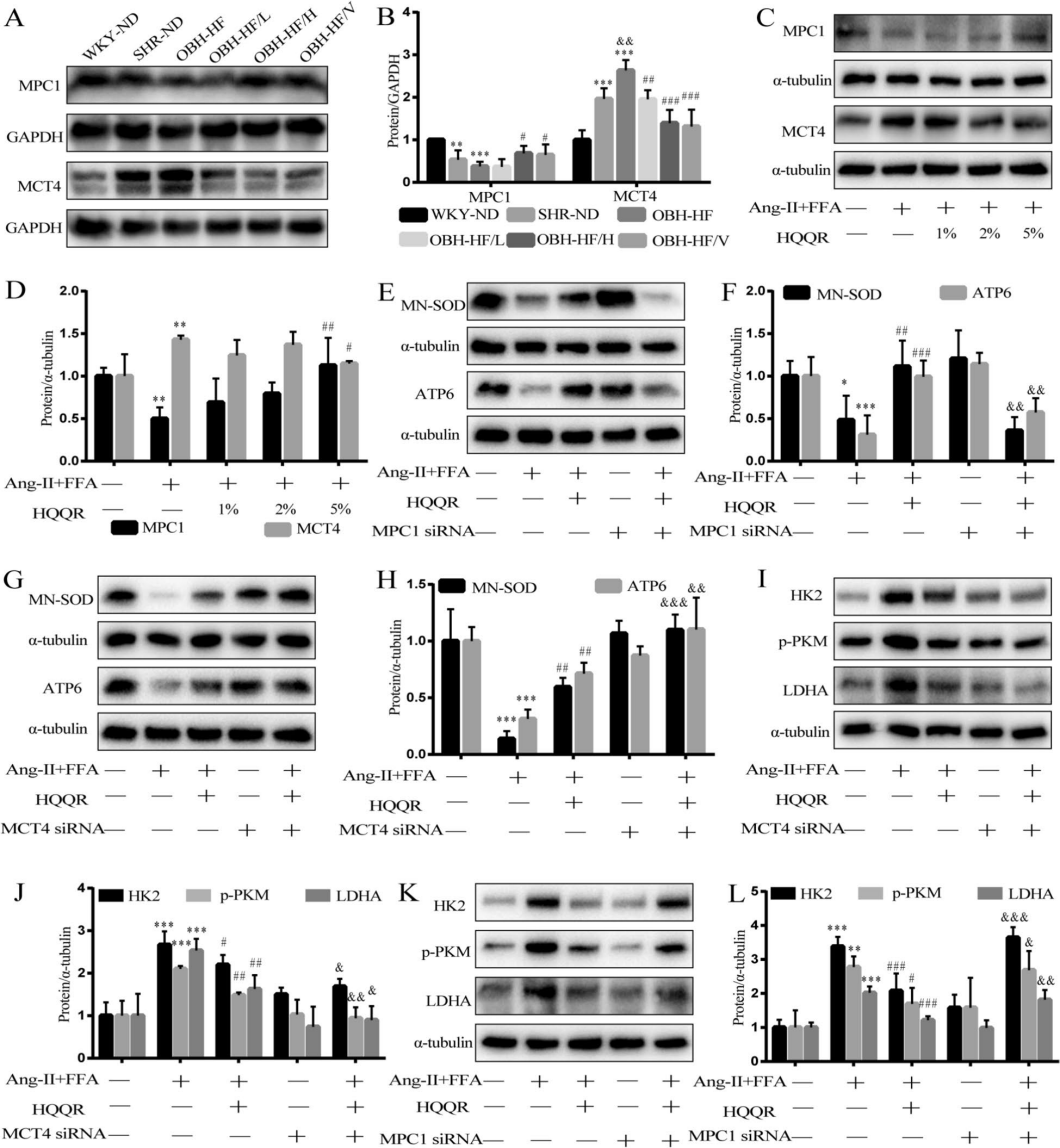
HQQR improves pyruvate-lactate metabolism and myocardial hypertrophy in vivo and in vitro by targeting MPC1/MCT4. **A** The MPC1 and MCT4 protein expression in myocardial tissue from each group of rats was tested by immunoblotting. **B** Quantitative analysis of MPC1 and MCT4 protein levels in myocardial tissue of rats (*n* = 4). **C** Representative immunoblot demonstrating MPC1 and MCT4 protein expression in H9C2 cells under different treatments. **D** Quantitative analysis of MPC1 and MCT4 proteins in H9C2 cells (*n* = 4). **E** Representative immunoblot demonstrating Mn-SOD and ATP6 protein expression in H9C2 cells under different treatments. **F** Quantitative analysis of Mn-SOD and ATP6 proteins in H9C2 cells (*n* = 4). **G** Representative immunoblot demonstrating Mn-SOD and ATP6 protein expression in H9C2 cells under different treatments. **H** Quantitative analysis of Mn-SOD and ATP6 proteins in H9C2 cells (*n* = 4). **I** Representative immunoblot demonstrating HK2, p-PKM and LDHA protein expression in H9C2 cells under different treatments. **J** Quantitative analysis of HK2, p-PKM and LDHA proteins in H9C2 cells (*n* = 4). **K** Representative immunoblot demonstrating HK2, p-PKM and LDHA protein expression in H9C2 cells under different treatments. **L** Quantitative analysis of HK2, p-PKM and LDHA proteins in H9C2 cells (*n* = 4). ^*^p < 0.05, ^**^p < 0.01, ^***^p < 0.001 *vs.* WKY-ND or control group, ^&^*p* < 0.05, ^&&^*p* < 0.01, ^&&&^*p* < 0.001 *vs.* SHR-ND or Ang-II + FFA + HQQR group, ^#^*p* < 0.05, ^##^*p* < 0.01, ^###^*p* < 0.001 *vs.* OBH-HF or Ang-II + FFA group

To investigate how HQQR modulates the pyruvate/lactate metabolic axis, we examined MPC1/MCT4 expression. Immunoblotting showed decreased MPC1 and increased MCT4 protein expression in the OBH-HF group versus SHR-ND or WKY-ND controls, which was reversed by HQQR treatment (Fig. 6A and B). In vitro, the combination of FFA and Ang-II further decreased MPC1 protein expression and increased MCT4 protein expression in H9C2 cells compared with Ang-II treatment alone (Supplementary Fig. 4). Of note, treatment with HQQR significantly inhibited the expression of MCT4 protein and up-regulated the expression of MPC1 protein in Ang-II + FFA-treated H9C2 cells (Fig. 6C and D). To determine whether HQQR improves mitochondrial function and metabolic axis balance via MPC1/MCT4 targeting, we transfected H9C2 cells with MPC1 siRNA or MCT4 siRNA. Consistent with previous results, HQQR upregulated Mn-SOD and ATP6 expression in wild-type H9C2 cells exposed to Ang-II + FFA. This effect was suppressed in MPC1-deficient cardiomyocytes but enhanced in MCT4-deficient cardiomyocytes (Fig. 6E–H). Furthermore, Western blotting showed that treatment with HQQR further reduced the content of HK2, p-PKM, and LDHA proteins in H9C2 cells of MCT4 knockdown under Ang-II + FFA stimulation (Fig. 6I and J). However, MPC1 siRNA transfection greatly attenuated the inhibition of glycolysis by HQQR (Fig. 6K and L).

**Fig. 7:**
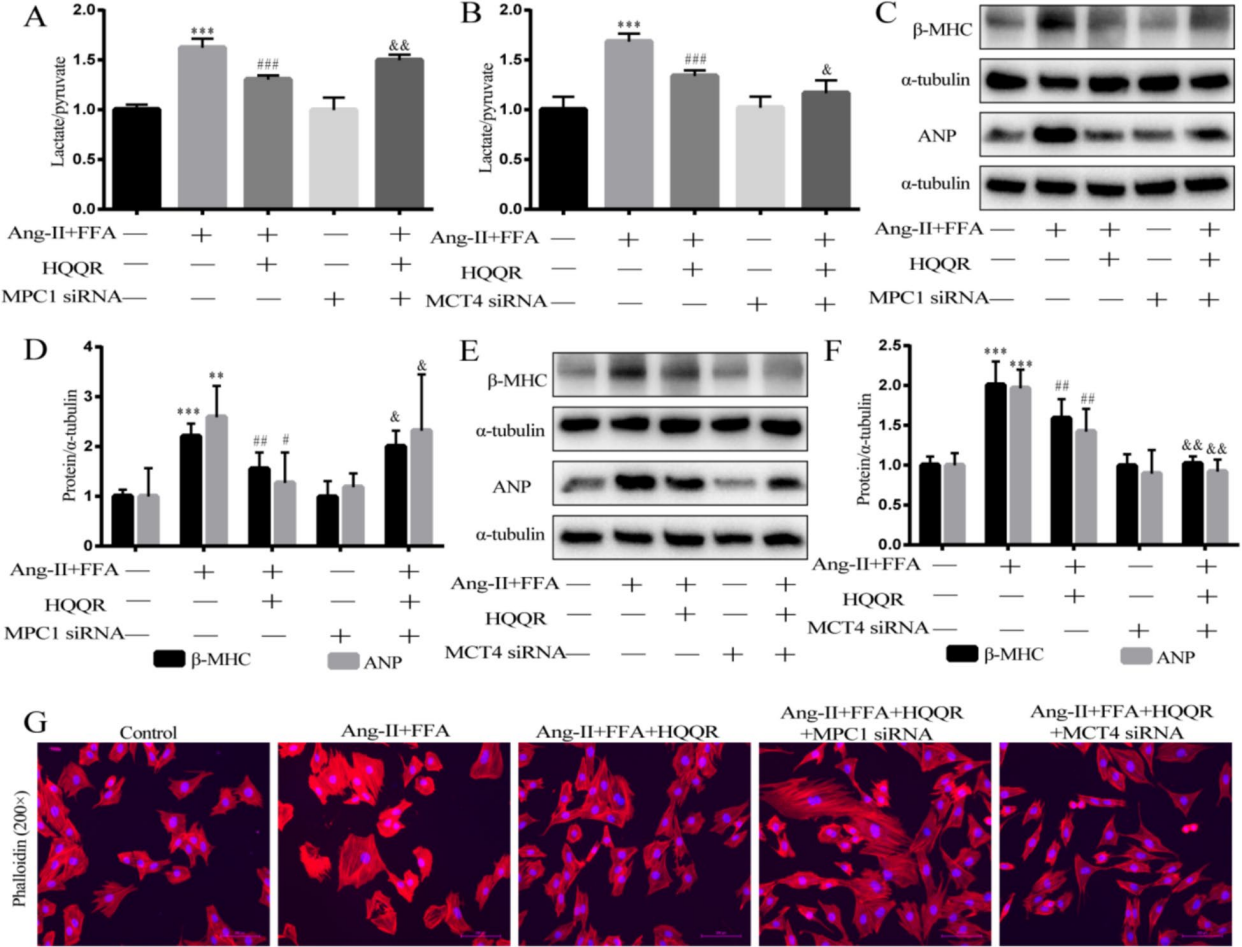
HQQR improves pyruvate-lactate metabolism and myocardial hypertrophy in vivo and in vitro by targeting MPC1/MCT4. **A**, **B** H9C2 cells were transfected with MPC1 siRNA or MCT4 siRNA, and after various treatments, lactate and pyruvate levels in the medium were measured and the ratio was calculated. **C** Representative immunoblot demonstrating β-MHC and ANP protein expression in H9C2 cells under different treatments. **D** Quantitative analysis of β-MHC and ANP proteins in H9C2 cells (*n* = 4). **E** Representative immunoblot demonstrating β-MHC and ANP protein expression in H9C2 cells under different treatments. **F** Quantitative analysis of β-MHC and ANP proteins in H9C2 cells (*n* = 4). **G** The morphology of H9C2 cells was observed by phalloidin staining using fluorescence microscopy at 200 × magnification. ^**^p < 0.01, ^***^p < 0.001 *vs.* control group, ^#^*p* < 0.05, ^##^*p* < 0.01, ^###^*p* < 0.001 *vs.* Ang-II + FFA group, ^&^*p* < 0.05, ^&&^*p* < 0.01 *vs.* Ang-II + FFA + HQQR group

More importantly, the ratio of lactate to pyruvate was positively correlated with the degree of glycolysis, which was reflected by an elevated ratio in MPC1 siRNA-transfected cells and a decreased ratio in MCT4 siRNA-transfected cells under HQQR treatment (Fig. 7A and B). Consistent with these results, in Ang-II + FFA-treated H9C2 cells, HQQR significantly reduced the fluorescence density of F-actin and decreased the expression of β-MHC and ANP proteins, which was suppressed in MPC1 gene-deficient cardiomyocytes and further enhanced in MCT4 gene-deficient cardiomyocytes. (Fig. 7C–G). These results suggests HQQR improves pyruvate-lactate metabolism and myocardial hypertrophy in Ang-II + FFA-stimulated H9C2 cells by targeting MPC1/MCT4.

### HQQR attenuates hypertrophy of lactate-stimulated H9C2 cells

**Fig. 8:**
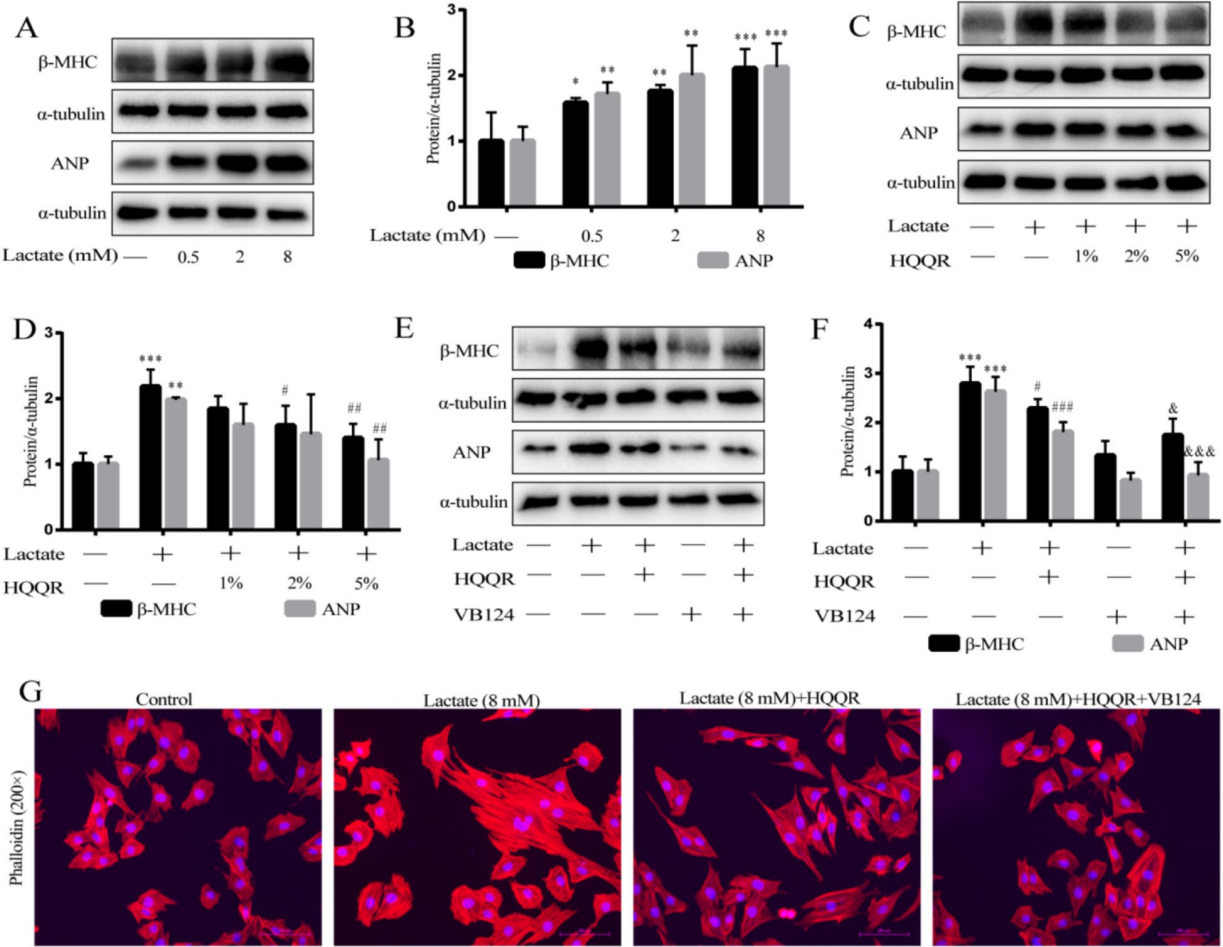
HQQR attenuates hypertrophy of lactate-stimulated H9C2 cells. **A** Representative immunoblot demonstrating β-MHC and ANP protein expression in H9C2 cells under different treatments. **B** Quantitative analysis of β-MHC and ANP proteins in H9C2 cells (*n* = 3). **C** Representative immunoblot demonstrating β-MHC and ANP protein expression in H9C2 cells under different treatments. **D** Quantitative analysis of β-MHC and ANP proteins in H9C2 cells (*n* = 4). **E** Representative immunoblot demonstrating β-MHC and ANP protein expression in H9C2 cells under different treatments. **F** Quantitative analysis of β-MHC and ANP proteins in H9C2 cells (*n* = 3). **G** The morphology of H9C2 cells was observed by phalloidin staining using fluorescence microscopy at 200 × magnification. ^*^p < 0.05, ^**^p < 0.01, ^***^p < 0.001 *vs.* control group, ^#^*p* < 0.05, ^##^*p* < 0.01, ^###^*p* < 0.001 *vs.* Lactate treatment group, ^&^*p* < 0.05, ^&&&^*p* < 0.001 *vs.* Lactate + HQQR group

Growing evidence has supported the role of lactate in inducing myocardial hypertrophy [33]. Given the apparent imbalance in pyruvate/lactate metabolism in OBH rats and Ang-II + FFA-treated H9C2 cells, we further determined whether extracellular lactate induces myocardial hypertrophy. Immunoblotting showed that lactate dose-dependently increased the expression of β-MHC and ANP proteins in H9C2 cells, indicating that lactate markedly induced the myocardial hypertrophy (Fig. 8A and B). Immunoblotting further demonstrated that HQQR-medicated serum inhibited hypertrophy in lactate-stimulated H9C2 cells concentration-dependently, evidenced by reduced β-MHC and ANP expression (Fig. 8C and D). To further determine whether HQQR inhibits lactate-mediated myocardial hypertrophy by down-regulating lactate transporter proteins, we treated H9C2 cells with VB124, a lactate transporter inhibitor. Consistent with the above results, HQQR significantly reduced the levels of β-MHC and ANP proteins in lactate-treated H9C2 cells (Fig. 8E and F). Notably, combining HQQR with VB124 further suppressed β-MHC and ANP expression compared to HQQR alone (Fig. 8E and F). As shown in Fig. 8G, lactate stimulation significantly induced hypertrophy of H9C2 cells, while HQQR treatment alleviated this hypertrophy, with particularly significant effects when used in combination with VB124. These findings suggest pyruvate/lactate dysregulation contributes significantly to myocardial hypertrophy and that HQQR's cardioprotective effects involve restoring pyruvate-lactate metabolic axis balance.

## Discussion

**Fig. 9:**
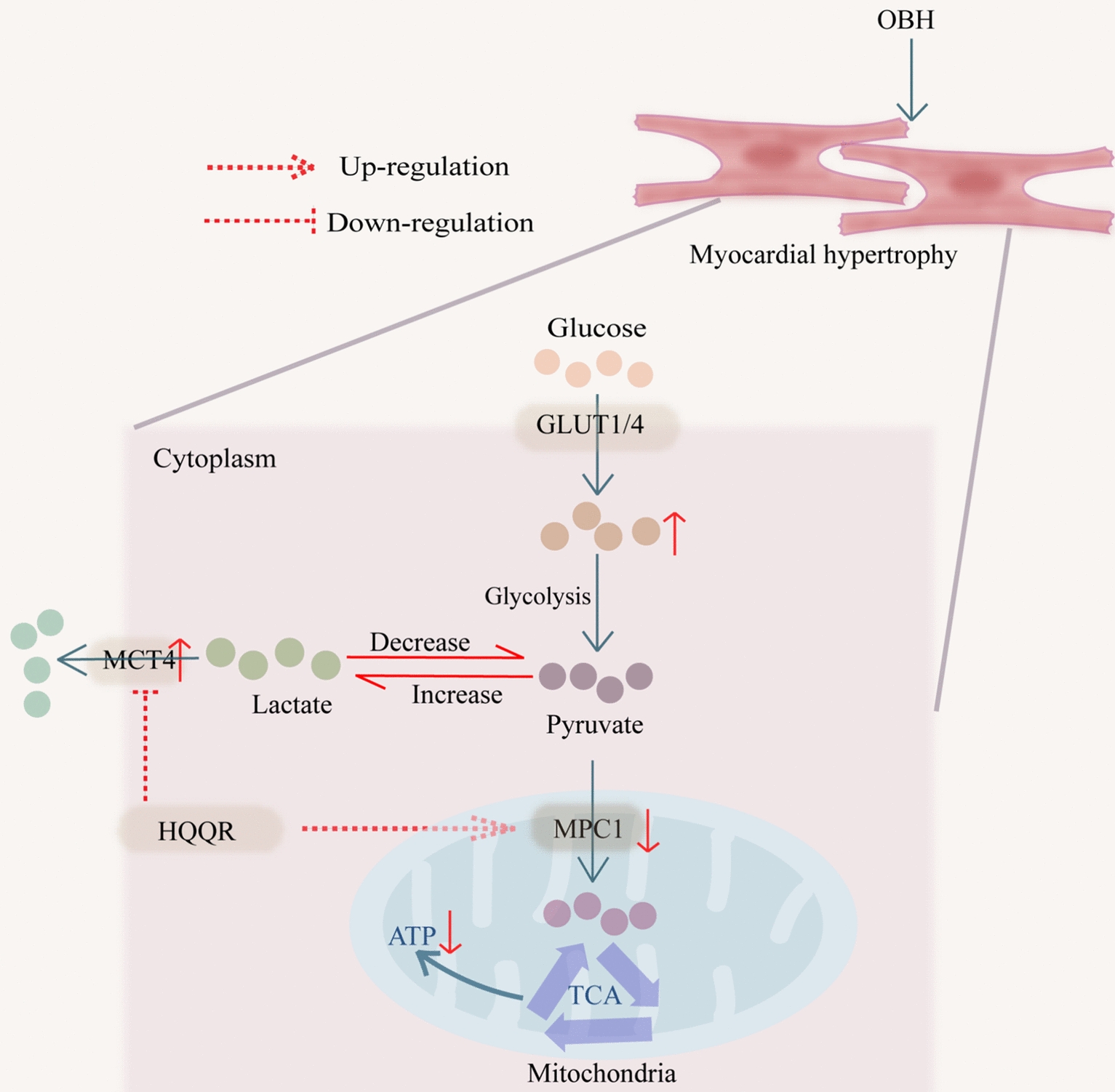
In pathological states, cardiomyocytes take up excess glucose and convert it to pyruvate via glycolysis. Pyruvate is preferentially converted to lactate rather than entering the mitochondria, thereby increasing lactate efflux. HQQR promotes the pyruvate oxidation and inhibits the lactate efflux by correcting the pyruvate-lactate axis via MPC1/MCT4, thus exerting an antimyocardial hypertrophic effect

In this study, we described the myocardial protective mechanism of HQQR in OBH rats, highlighting the key role of the MPC1/MCT4-mediated pyruvate/lactate metabolic axis in myocardial hypertrophy (Fig. 9). We identified obesity as a risk factor for exacerbating myocardial hypertrophy, inducing more pronounced mitochondrial damage and glucose metabolism imbalance. From a therapeutic perspective, we determined that HQQR dose-dependently improved myocardial hypertrophy, mitochondrial function, glycolysis, and pyruvate/lactate metabolism in OBH rats and Ang-Ⅱ combined with FFA-treated H9C2 cells. Of note, using siRNA transfection approaches, we further demonstrated that MPC1/MCT4 is the potential targets of HQQR to attenuate myocardial hypertrophy in OBH rats. Finally, we found that lactate dose-dependently induced myocardial hypertrophy, suggesting that lactate is not only a metabolite but also a pathological factor that induces myocardial remodelling.

An imbalance in the pyruvate-lactate metabolic axis represents an early feature of myocardial hypertrophy [34, 35]. During pathological myocardial remodelling, oxidative metabolism of glucose in mitochondria does not match glycolysis, leading to excessive conversion of pyruvate to lactate. Growing evidence indicates that lactate accumulation and efflux are closely associated with pathological myocardial remodeling. Zhao et al. reported that inhibition of glycolysis or reduction of lactate generation significantly attenuated the myocardial hypertrophy by inhibiting histone lysine lactylation in transverse aortic constriction surgery-established myocardial hypertrophy mice [36]. Wei et al. found that elevated lactate production by Ang-Ⅱ-treated cardiac fibroblasts induced marked cardiomyocyte hypertrophy via MCT1-mediated lactate inputs [37]. In the present study, we demonstrated that OBH rats or Ang-Ⅱ combined with FFA-treated cardiomyocytes exhibited significantly mitochondrial dysfunction and abnormally elevated lactate/pyruvate ratio, suggesting an imbalance between glycolysis and pyruvate oxidation. More importantly, HQQR has the potential to correct glucose metabolism and restores mitochondrial homeostasis in cardiomyocytes. Therefore, our studies suggested that restoration of pyruvate oxidation and reduction of lactate production were key mechanisms by which HQQR exerts its effects against myocardial hypertrophy. In addition, given the key role of lactate in myocardial hypertrophy, we further explored whether HQQR could attenuate lactate-induced myocardial injury. Our results demonstrated that HQQR treatment significantly decreased the expression of β-MHC and ANP proteins in lactate-stimulated H9C2 cells, which was further inhibited by the combined use of VB124. These findings also suggest that lactate functions are not merely as a glycolytic end-product but as a key mediator of myocardial hypertrophy.

To explore HQQR's regulatory mechanisms on this metabolic axis, we focused on MPC1/MCT4. MPC1 is a key transporter linking glycolysis and mitochondrial oxidative phosphorylation [38]. MPC1 can transport pyruvate, a product of glycolysis, to mitochondria where it participates in oxidative metabolism through the tricarboxylic acid cycle [39, 40]. In the process of myocardial hypertrophy, mitochondrial dysfunction and reduced MPC1 expression impede the entry of pyruvate into the mitochondria, which is then converted to lactate by compensatory energy supply through glycolysis. The previous studies reported that loss of MPC1 activity directly altered pyruvate metabolism in mitochondria, and resulted in excessive lactate production and efflux. Zhang and colleagues found that cardiomyocyte-restricted MPC1-knockout mice developed age-dependent pathological myocardial hypertrophy, ultimately leading to premature death [14]. Meanwhile, MPC1 overexpression attenuated drug-induced myocardial hypertrophy by increasing the flux of pyruvate into the mitochondria [41]. MCT4 is the major lactate transporter in cardiomyocytes [42]. Studies have shown that elevated MCT4 expression leads to increased lactate efflux and the inability to reverse lactate to pyruvate, leading to disturbances in the pyruvate-lactate metabolic axis [15, 43]. In addition, inhibition of MCT4 has been shown to improve the hypertrophy of mouse cardiomyocytes by restoring pyruvate flux and improving oxidative stress in the cytoplasm [34]. Furthermore, the vicious cycle between MPC1/MCT4-mediated imbalance of the pyruvate-lactate metabolic axis and mitochondrial dysfunction accelerates the development of myocardial hypertrophy [44]. Our previous studies found the significant mitochondrial structural damage and dysfunction in myocardial tissues of OBH rats, and HQQR improved mitochondrial function and morphology in OBH rats [28]. We fed SHR rats with a HFD to construct OBH and used Ang-Ⅱ + FFA to induce myocardial hypertrophy. Our results suggested that high expression of MCT4 protein and low expression of MPC1 protein in myocardial tissues of OBH rats was closely related to imbalance of the pyruvate/lactate metabolic axis and mitochondrial dysfunction. From a therapeutic perspective, HQQR restored the pyruvate/lactate metabolic axis in OBH rats and Ang-Ⅱ + FFA-treated H9C2 cells by decreasing the MCT4 protein level and elevating the MPC1 protein level, thus exerting an anti-myocardial hypertrophic effect. These findings establish HQQR as a promising therapeutic agent for myocardial hypertrophy through modulation of the MPC1/MCT4-mediated pyruvate-lactate metabolic axis.

However, this study still has some limitations. Firstly, we have yet to investigate in depth the specific molecular mechanisms by which HQQR regulates MPC1/MCT4. On the one hand, as this study reveals, HQQR contains multiple active constituents, among which there may exist molecules capable of directly binding to MPC1 or MCT4 and thereby regulating their function. On the other hand, HQQR may exert its cardioprotective effects by influencing molecular pathways associated with MPC1/MCT4. Subsequently, further investigations will be conducted using methods such as proteomic sequencing, molecular docking, and drug affinity responsive target stability, etc. Secondly, we only included valsartan as a positive control in animal experiments, but not in cell experiments. The use of valsartan in cellular experiments would better elucidate the cardioprotective effects of HQQR. Thirdly, we primarily employed inhibitors and siRNA to verify that HQQR exerts its cardioprotective effects though MPC1/MCT4-mediated pyruvate/lactate axis. However, validation using overexpression methods would render our conclusions more compelling.

## Conclusion

Collectively, we demonstrate that HQQR is a viable drug candidate for treating myocardial hypertrophy in OBH, including the reduction of blood pressure, lipids and lactate/pyruvate ratio. Mechanistically, we identify that HQQR restores mitochondrial homeostasis and balance of the pyruvate/lactate metabolic axis by inhibiting MCT4 and up-regulating MPC1, thereby ameliorating cardiomyocyte injury and slowing the progression of OBH.

## Supplementary Information

Supplementary Material 1.

Supplementary Material 2.

Supplementary Material 3.

## Data Availability

The datasets used and/or analysed during the current study are available from the corresponding author on reasonable request.

## Abbreviations

Ang-II: Angiotensin II
ANP: Atrial natriuretic peptide
β-MHC: β-Myosin heavy chain
FFA: Free fatty acid
GLUT1: Glucose transporter-1
HE: Hematoxylin & eosin
HFD: High-fat diet
HK2: Hexokinase 2
HQQR: HuoXue QianYang QuTan recipe
LDHA: Lactate dehydrogenase
MCT4: Monocarboxylate transporter 4
MPC1: Mitochondrial pyruvate carrier 1
OBH: Obesity hypertension
SHR: Spontaneously hypertensive rats
TCM: Traditional Chinese medicine
WGA: Wheat germ agglutinin
