# Supplementary material for: HuoXue QianYang QuTan recipe attenuates myocardial hypertrophy in obese hypertensive rats by regulating MPC1/MCT4 mediated pyruvate-lactate metabolic axis
Source: Chin Med. 2025 Oct 23;20:180. doi: 10.1186/s13020-025-01240-9 (PMC12548125; doi:10.1186/s13020-025-01240-9)
Supplement: Supplementary file 1 — Supplementary Material 1. [file 13020_2025_1240_MOESM1_ESM.docx]

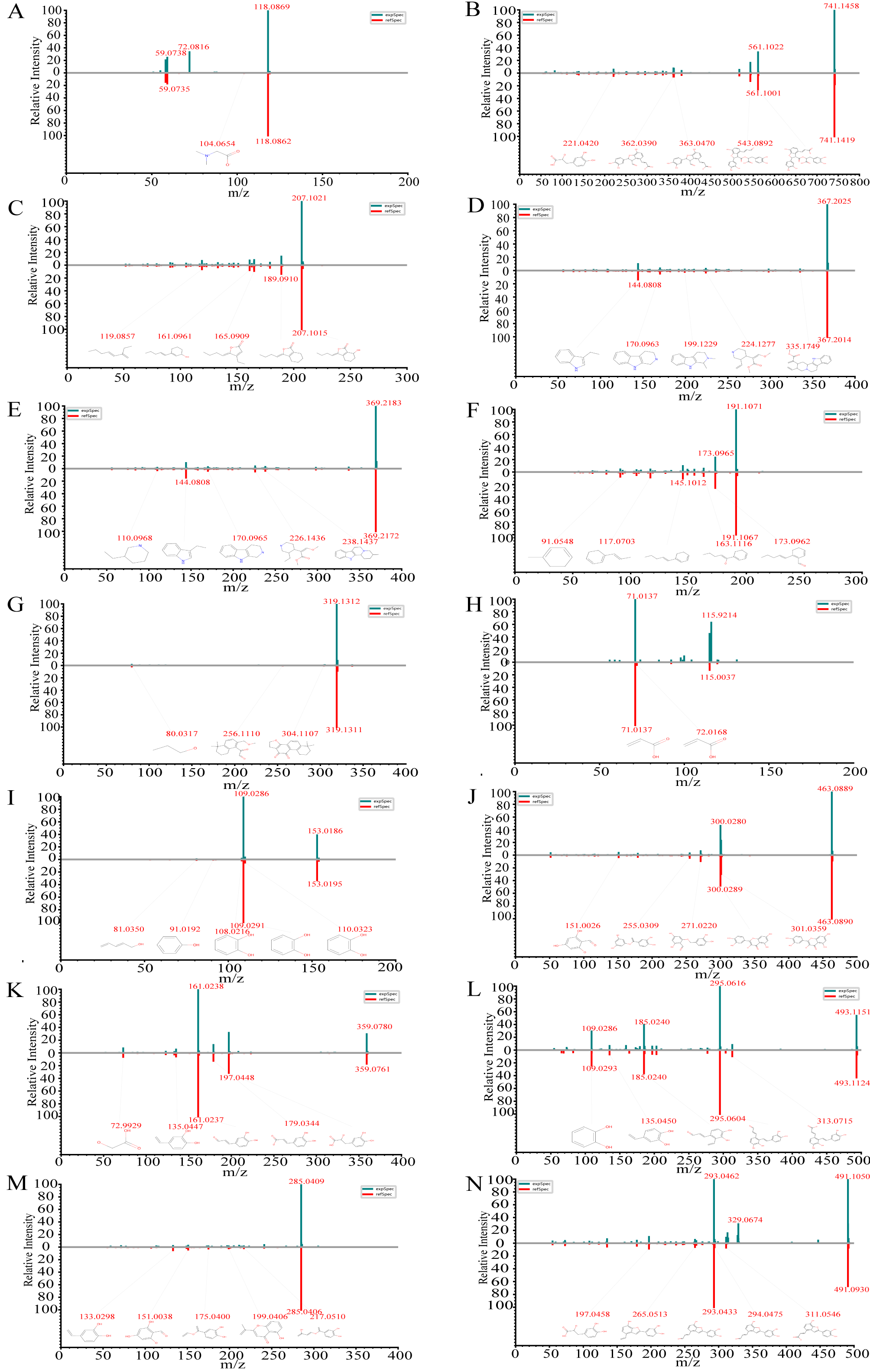


**Supplemental Fig. 1.** UPLC-MS was used to analyse the chemical composition of HQQR and obtain the Base-peak chromatogram (BPC) of HQQR. Ion chromatograms of Betaine (**A**), Salvianolic acid B (**B**), Senkyunolide I (**C**), Hirsuteine (**D**), Hirsutine (**E**), Ligusticide (**F**), Cryptotanshinone (**G**), Fumaric acid (**H**), Protocatechuic acid (**I**), Isoquercitrin (**J**), Rosmarinic acid (**K**), Salvianolic acid A (**L**), Luteolin (**M**), Salvianolic acid C (**N**).


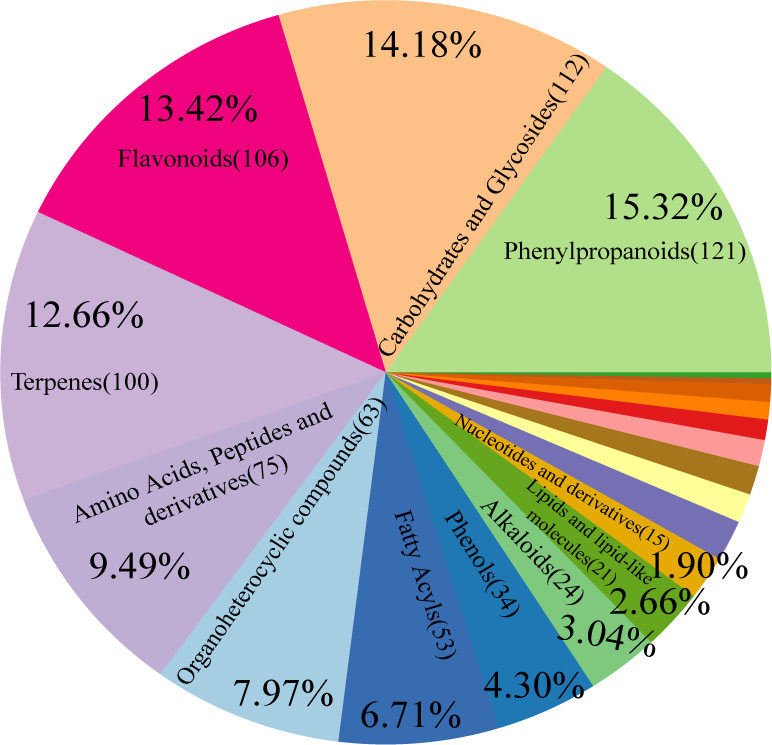


**Supplemental Fig. 2.** Classification diagram of components identified in HQQR.


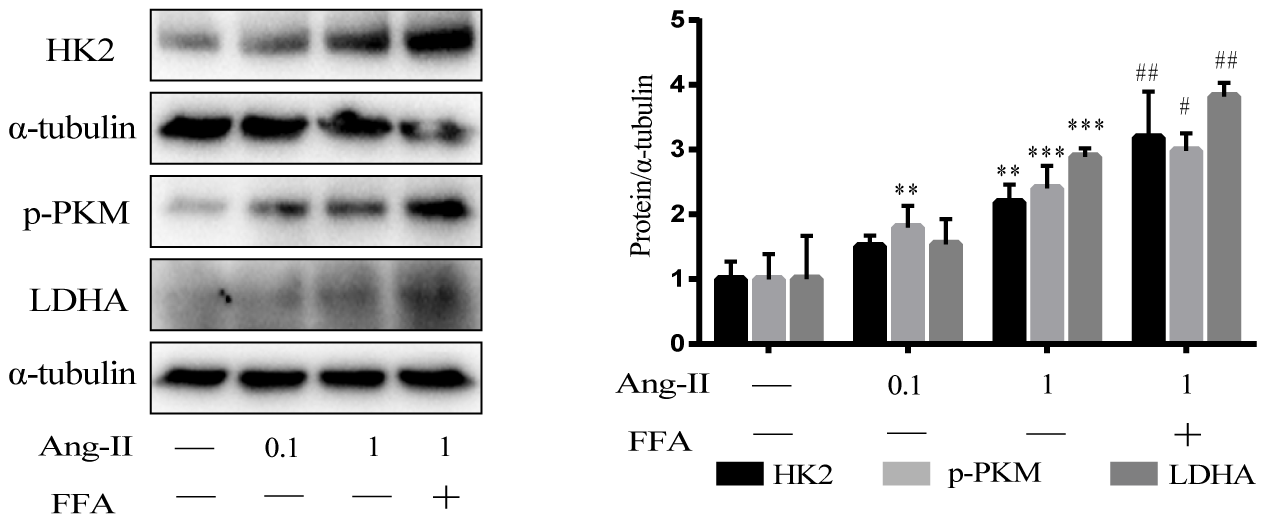


**Supplemental Fig. 3.** The H9C2 cells were treated with Ang-Ⅱ (0.1 μM or 1 μM) in the presence or absence of FFA. The HK2, p-PKM and LDHA protein content in H9C2 cells was demonstrated by immunoblotting and subsequently quantified. ^**^*p* < 0.01, ^***^*p* < 0.001 *vs.* control group, ^#^*p* < 0.05, ^##^*p* < 0.01 *vs.* Ang-Ⅱ (1 μM) treatment group.


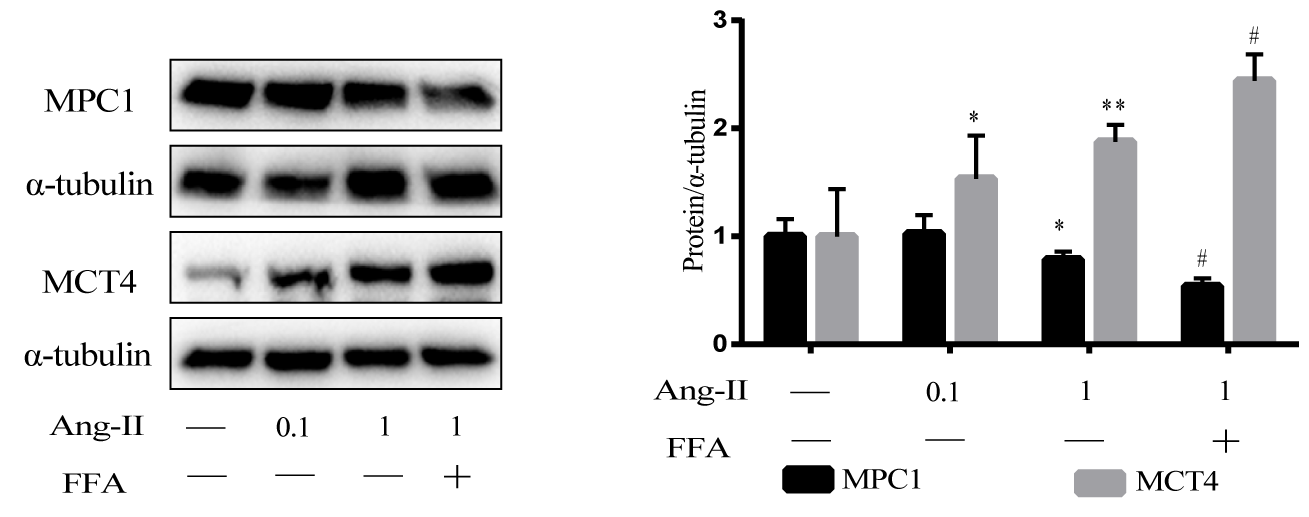


**Supplemental Fig. 4.** The H9C2 cells were treated with Ang-Ⅱ (0.1 μM or 1 μM) in the presence or absence of FFA. The MPC1 and MCT4 protein expression in H9C2 cells was demonstrated by immunoblotting. ^*^*p* < 0.05, ^**^*p* < 0.01 *vs.* control group, ^#^*p* < 0.05 *vs.* Ang-Ⅱ (1 μM) treatment group.
