# Supplementary material for: HuoXue QianYang QuTan recipe attenuates myocardial hypertrophy in obese hypertensive rats by regulating MPC1/MCT4 mediated pyruvate-lactate metabolic axis
Source: Chin Med. 2025 Oct 23;20:180. doi: 10.1186/s13020-025-01240-9 (PMC12548125; doi:10.1186/s13020-025-01240-9)
Supplement: Supplementary file 2 — Supplementary Material 2. [file 13020_2025_1240_MOESM2_ESM.docx]

| Binominal name | Family | Genus | Species | Parts used | Proportion (g) |
| --- | --- | --- | --- | --- | --- |
| *Salvia miltiorrhiza* Bge. | Lamiaceae | *Salvia Linn* | *S. miltiorrhiza* | Root | 15 |
| *Ligusticum chuanxiong* Hort. | Apiaceae | *Ligusticum L.* | *Apiales* | Dry rhizome | 9 |
| *Uncaria rhynchophylla* (Miq.) Miq. ex Havil. | Asteraceae | *Echinacea* | *E. angustifolia* | Dry with hook stem branches | 15 |
| *Haliotis diversicolor* Reeve | Haliotidae | *Haliotis* | *Concha haliotidis* | The shell of abalone | 30 |
| *Taxillus chinensis*(DC.) Danser | Loranthaceae | *Taxillus Van Tiegh* | *Parasitic loranthus* | Dry leafy stems and branches | 15 |
| *Crataegus pinnatifida* Bge. | Rosaceae | *Crataegus* | *Crataegus rhipidophylla* | Dry ripe fruit | 15 |
| *Zea mays* L. | Poaceae | *Zea* | *Zea mays L.* | Corn Stigma | 30 |

**Table 1.**

**Composition of HuoXue QianYang QuTan Recipe.**
